# Safety of Cold Snare Polypectomy during Continuous Use of Antithrombotic Drugs for Delayed Post-Polypectomy Bleeding: A Pilot Study

**DOI:** 10.1159/000531061

**Published:** 2023-05-15

**Authors:** Junnosuke Hayasaka, Satoshi Yamashita, Akira Matsui, Yusuke Kawai, Yorinari Ochiai, Takayuki Okamura, Yugo Suzuki, Yutaka Mitsunaga, Kosuke Nomura, Masami Tanaka, Kazuhiro Fuchinoue, Hiroyuki Odagiri, Daisuke Kikuchi, Yutaka Takazawa, Shu Hoteya

**Affiliations:** ^a^Department of Gastroenterology, Toranomon Hospital, Tokyo, Japan; ^b^Department of Pathology, Toranomon Hospital, Tokyo, Japan

**Keywords:** Cold snare polypectomy, Delayed post-polypectomy bleeding, Antithrombotic drug, Colonoscopy, Colon polyp

## Abstract

**Background:**

Cold snare polypectomy is a high-risk endoscopic procedure with a low delayed post-polypectomy bleeding rate. However, it is unclear whether delayed post-polypectomy bleeding rates increase during continuous antithrombotic treatment. This study aimed to determine the safety of cold snare polypectomy during continuous antithrombotic treatment.

**Methods:**

This single-center, retrospective cohort study enrolled patients who underwent cold snare polypectomy during antithrombotic treatment between January 2015 and December 2021. Patients were divided into continuation and withdrawal groups based on whether they continued with antithrombotic drugs or not. Propensity score matching was performed using age, sex, Charlson comorbidity index, hospitalization, scheduled treatment, type of antithrombotic drugs used, multiple medications used, indication for antithrombotic drugs, and gastrointestinal endoscopist qualifications. The delayed polypectomy bleeding rates were compared between the groups. Delayed polypectomy bleeding was defined as the presence of blood in stools and requiring endoscopic treatment or a decrease in hemoglobin level by 2 g/dL or more.

**Results:**

The continuation and withdrawal groups included 134 and 294 patients, respectively. Delayed polypectomy bleeding was observed in 2 patients (1.5%) and 1 patient (0.3%) in the continuation and withdrawal groups, respectively (*p* = 0.23), before propensity score matching, with no significant difference. After propensity score matching, delayed polypectomy bleeding was observed in 1 patient (0.9%) in the continuation group but not in the withdrawal group, with no significant difference.

**Conclusion:**

Cold snare polypectomy during continuous antithrombotic treatment did not significantly increase delayed post-polypectomy bleeding rates. Therefore, this procedure may be safe during continuous antithrombotic treatment.

## Introduction

In 2020, there are estimated to be more than 1.9 million new cases of colorectal cancer and 935,000 deaths [1]. Overall, colorectal cancer ranked third in incidence and second in mortality in 2020 [1]. Therefore, it is important to reduce the number of colorectal cancer-related deaths. Colorectal polypectomy is widely performed as it has been reported to reduce mortality from colorectal cancer [2−4]. However, a major adverse event of polypectomy is post-polypectomy bleeding, the frequency of which has been reported to be approximately 1% [5]. Cold snare polypectomy (CSP) has been introduced as a safe and effective method for removing polyps smaller than 10 mm [6]. CSP has fewer adverse events than other polypectomy techniques [7, 8], and it is now widely practiced. Increased delayed post-polypectomy bleeding (DPPB) rate in CSP has been reported in patients using antithrombotic drugs [9−11], and in such cases, CSP may not always be safe. As the number of patients taking antithrombotic drugs is increasing owing to the aging population [12, 13], CSP is also being increasingly performed on those on antithrombotic medications. Therefore, appropriate management of antithrombotic drugs is necessary.

CSP is a high-risk endoscopic procedure according to the Japan Gastroenterological Endoscopy Society (JGES), American Society for Gastrointestinal Endoscopy (ASGE), and European Society of Gastrointestinal Endoscopy (ESGE) guidelines, and it requires the appropriate withdrawal of antithrombotic drugs depending on the type [14−16]. However, withdrawal of antithrombotic drugs carries the risk of developing thromboembolism. Furthermore, thromboembolism, once developed, usually results in a fatal course and sequelae. Therefore, if DPPB does not increase, CSP during continuous antithrombotic treatment is preferable. Furthermore, performing CSP alongside continuous antithrombotic treatment has the potential benefit of eliminating the need for repeat colonoscopy to withdraw the antithrombotic drugs. The continuation of aspirin alone has been considered safe for many endoscopic procedures [14−16]. There are limited reports of CSP during continuous antithrombotic treatment; however, a report showed an increase in DPPB with direct oral anticoagulants (DOACs) [17], whereas no increase was observed under dual antiplatelet therapy (DAPT) [18, 19]. Therefore, it is unclear whether the risk of DPPB increases with CSP when antithrombotic drugs are continued or withdrawn. Furthermore, there are limited reports of using CSP in real-world data (RWD). Therefore, this study retrospectively investigated the rate of DPPB after CSP during continuous antithrombotic treatment using RWD.

## Materials and Methods

We conducted a single-center, retrospective cohort study. In total, 3,486 consecutive patients underwent CSP between January 2015 and December 2021. Of these, 427 patients who received antithrombotic drugs were enrolled in this study. The patients were divided into continuation and withdrawal groups based on whether they continued using antithrombotic drugs or not. Antithrombotic drugs in this study included antiplatelet (aspirin, thienopyridines, and cilostazol) and anticoagulant (warfarin, DOACs, and Fragmin) drugs. The thienopyridine included ticlopidine, clopidogrel, and prasugrel. The DOACs included dabigatran, rivaroxaban, apixaban, and edoxaban.

The study was approved by the Toranomon Hospital Ethics Committee (approval number: 2313) and conducted in accordance with the tenets of the 1975 Declaration of Helsinki (6th edition, 2008). Patients' informed consent was obtained through an opt-out method.

### Clinical Data Assessment

We collected data on age, sex, Charlson comorbidity index (CCI) [20], which has been widely used and validated for use in gastrointestinal bleeding research [21−23], type of antithrombotic drugs used, indications for antithrombotic drugs, continued antithrombotic drugs, replacement of drugs, endoscopic lesion factors (size, location, and morphology), pathological diagnosis, additional hemostatic procedures, and gastrointestinal endoscopists. The polyp locations were divided into right-sided colon (from the cecum to the transverse colon) and left-sided colon (from the descending colon to the rectum). Polyp shapes were divided into polypoid and non-polypoid lesions according to the Paris classification [24]. Polyp sizes were measured using snares. The continuation group included those with an absence of antithrombotic drug withdrawal, whereas the withdrawal group included those with replaced antithrombotic drugs. The final decision on antithrombotic drug withdrawal was made by the doctor in charge after consultation with an expert antithrombotic prescribing physician with reference to the current guidelines [14, 25] and considering the patient's preferences and condition.

### Procedure

The indication for CSP was non-pedunculated polyps ≤10 mm with suspected tumor and no findings of suspected cancer. However, resection was sometimes performed at the discretion of the physician. Most patients were administered 10 mL of sodium picosulfate the day before the colonoscopy and polyethylene glycol in the morning on the day of the examination.

In most cases, CSP was performed using a PCF-H290ZI or PCF-H290I colonoscope (Olympus Medical Systems, Tokyo, Japan). Otherwise, CSP was performed using other techniques (PCF-Q260AZI, CF-Q260AI, PCF-H290TI, PCF-PQ260L, PCF-PQ260I, SIF-H290S, PCF-Q260JI, CF-H290ECI, CF-XZ1200I, PCF-Q240ZI, CF-H290I, and CF-EZ1500DI [Olympus Medical Systems, Tokyo, Japan]; EC-L600ZP7; and FUJIFILM, [Saitama, Japan]) were used for CSP. In all cases, the colonoscope was fitted with a soft cap, and where possible, a water-jet device was used. In most cases, a cold snare (Micro-Tech Co. Ltd., Nanjing, China) was used, and in some cases, a Snare Master 10 mm (Olympus, Tokyo, Japan) was used. For complete resection, polyps were resected with surrounding normal tissue, wherever possible. The resected polyps were collected using gauze through the suction channel of the colonoscope. Clipping was performed in cases of immediate bleeding or when deemed necessary by the endoscopist. If DPPB was not observed after CSP, the patient was instructed to resume antithrombotic drugs the following day.

### Outcomes

The primary endpoint was DPPB. Secondary endpoints were minor bleeding, thromboembolism, and perforation. DPPB was defined as the presence of blood in stools within 14 days after treatment and requiring endoscopic treatment or a decrease in hemoglobin (Hb) by 2 g/dL or more. Secondary endpoints were cases in which the patient was investigated for the main complaint of bloody stools that occurred up to 14 days after treatment. Minor bleeding was defined as an unscheduled consultation, hospital admission, or colonoscopy, none of which required endoscopic treatment. Thromboembolism included cases of cerebral ischemic disease, ischemic heart disease, pulmonary embolism, and deep vein thrombosis.

### Statistical Analyses

Continuous and non-normally distributed variables are expressed as medians and interquartile ranges. They were compared using an unpaired *t* test or the Wilcoxon rank-sum test. CCI and the number of polyps that have a non-normal distribution were compared using the Wilcoxon rank-sum test. Categorical variables were compared using the χ^2^ test or Fisher's exact test. No data were missing. Propensity scores were calculated using logistic regression analysis, and the scores were estimated using the following variables: age, sex, hospitalization, scheduled treatment, antithrombotic drugs (aspirin, thienopyridine, cilostazol, warfarin, and DOAC), multiple medication use, indication for antithrombotic drugs, gastrointestinal endoscopist qualification, and CCI. Propensity score matching (PSM) was then performed using nearest neighbor matching and a caliper width of 0.2 for pooled standard deviations.

Statistical significance was set at *p* < 0.05. All analyses were performed using the R software version 4.1.0 (The R Foundation for Statistical Computing, Vienna, Austria). PSM was performed using *MatchIt* package (v4.9.9).

## Results

There were 134 and 293 patients in the continuation and withdrawal groups, respectively. Table 1 summarizes the background of both groups before and after PSM.

The continuation group was significantly more likely to take antiplatelet drugs (aspirin and cilostazol) than the withdrawal group. In contrast, the continuation group received significantly fewer anticoagulants and DOACs than the withdrawal group. Furthermore, there were significantly fewer multiple medications administered in the continuation group than in the withdrawal group. The indication for antithrombotic drugs was significantly less atrial fibrillation and more ischemic heart disease in the continuation group compared with the withdrawal group. In the PSM, 116 patients were matched for each group. After PSM, absolute standardized differences were less than 0.2 for all variables between the two groups.

Table 2 presents the characteristics of the polyps in each group. A total of 266 and 683 polyps were resected in the continuation and withdrawal groups, respectively, with a median of two polyps resected per patient in both groups. There were no significant differences in the preoperative lesion size, specimen size, location, morphology, or pathological diagnosis between the two groups.

The number of days of withdrawal and days between treatment and resumption in the withdrawal group are listed in Table 3. The median duration of withdrawal was 5 days for aspirin, 7 days for the thienopyridines, 2 days for cilostazol, 5 days for warfarin, 1 day for DOACs, and 1 day for Fragmin. The median time to restart was 1 day.

Table 4 presents the study outcomes. There was no significant difference in DPPB between both groups before PSM (1.5% and 0.3% in the continuation and withdrawal groups, respectively). Furthermore, there was no significant difference in DPPB between both groups after PSM (0.9% and 0% in the continuation and withdrawal groups, respectively). In the continuation group, 1 patient with DPPB was treated with aspirin and another with a thienopyridine. One patient in the withdrawal group who developed DPPB was on thienopyridine and DOAC. The thienopyridine was withdrawn for 7 days and DOAC for 1 day and resumed 1 and 2 days later, respectively; however, the patient developed DPPB on day 3 after CSP. Minor bleeding before and after PSM was not significantly different between the two groups. Thromboembolism occurred only in the withdrawal group, with a frequency of 0.7%, although there were no significant differences. There was thromboembolism in 2 cases: a patient on DAPT developed acute coronary syndrome during endoscopic treatment after a thienopyridine was withdrawn for 10 days. The other patient on DOAC for atrial fibrillation developed transient ischemic attack on day 2 after CSP after DOAC was withdrawn for 2 days and resumed the day after treatment. There were no significant differences in other outcomes between the two groups. After PSM, no significant differences were observed in any of the secondary outcomes.

## Discussion

This study retrospectively investigated the safety of CSP with continued antithrombotic drug administration using RWD. No increase in DPPB rate was observed in CSP under continuous antithrombotic drug administration, even after PSM. Furthermore, no increase in any other adverse event was observed. Therefore, CSP can be safely performed with continuous antithrombotic treatment.

In the continuation group, DPPB rate was 1.5% before PSM and 0.9% after PSM. There was no increase in DPPB rate compared to the withdrawal group. One reason for the lack of an increase in DPPB rate may be because antiplatelet drugs account for approximately 75% of antithrombotic drugs. Continuous use of aspirin has been reported to be safe for many endoscopic procedures [14−16]. The ESGE guidelines consider the continuation of clopidogrel in CSP for polyps <10 mm [16]. Furthermore, DPPB rate in CSP with the continuous use of DAPT ranged from 1.7% to 2.4%, with no significant increase compared with the withdrawal group [18, 19]. Therefore, it is assumed that continuing antiplatelet drugs in CSP does not increase DPPB rate.

Anticoagulant use has been reported as a risk factor for DPPB in CSP [11]. However, the DPPB rate of CSP with continuous warfarin administration was reported to be 0% (0/35) [8]. Similarly, in this study, DPPB rate was 0% (0/12) with continuous warfarin administration, and thus, CSP with continuous warfarin administration may not increase DPPB. DPPB rate was reported to increase by 8.5% (4/47) in the CSP group with continuous DOAC administration compared to the withdrawal group [17]; however, in this study, no increase in DPPB rate with continuous DOAC administration (0%, 0/19) was observed. These differences may be due to the fact that the previous report used the DOAC continuation group as a historical control [17], which may have resulted in a higher DPPB rate.

Therefore, the study period was the same, and DPPB may not increase under continuous DOAC administration. However, neither our report nor previous reports [17] have used a large sample size with continuous DOAC administration; hence, more cases need to be investigated in the future.

Other reasons for the lack of an increase in the DPPB rate in the continuation group may have been the inclusion of hospitalized patients or high clipping rate. The DPPB rate may not have increased during hospitalization, as patients were considered more rested than in an outpatient setting due to more restricted behavior such as exercise or work. Immediate bleeding could not be assessed in this study; however, the clipping rate was high, exceeding 60% in both groups. As immediate bleeding rates of 4.8–14.2% have been reported in CSP under continuous use of antithrombotic drugs [17−19], it is assumed that many of the clipping cases in this study were prophylactic clippings, which may have affected the DPPB rate. Therefore, DPPB may be prevented by choosing appropriate hospitalization treatment or clipping, even in CSP, with continuous antithrombotic drug administration.

The frequency of minor bleeding was low and did not differ between the continuous and withdrawal groups. Minor bleeding has an impact on the patient, doctor burden, and healthcare costs. It was estimated that minor bleeding would be more common in the continuation group, although no increase was observed in this study. However, previous reports have shown less minor bleeding in CSP with continuous antithrombotic drug administration [19]. It was speculated that this was because of the prompt increase in immediate bleeding rates and clipping during continued antithrombotic treatment and that the resulting clipping may have prevented DPPB. Therefore, the high clipping rate in this study may have influenced the low DPPB rate with continuous antithrombotic drug administration. In addition, compared with outpatient cases, hospitalized cases may have been influenced by the increased physical rest and ability of the medical staff to check for bleeding, which determined whether follow-up could be performed without colonoscopy. However, minor bleeding may be unavoidable in outpatient treatment because only the patient can confirm the occurrence of bleeding. Therefore, minor bleeding may be prevented by choosing hospitalization or clipping, even with continuous antithrombotic therapy.

Withdrawal of antithrombotic drugs carries the risk of developing thromboembolism. In this study, 2 cases of thromboembolism only occurred in the withdrawal group with no statistically significant difference. One patient on DAPT was taken off a thienopyridine for only 10 days before CSP. According to PARIS (patterns of nonadherence to antiplatelet regimens in stented patients) registry 1, the adjusted hazard ratio for major adverse events (composite of cardiac death, definite or probable stent thrombosis, myocardial infarction, or target-lesion revascularization) when antiplatelet therapy was briefly interrupted was 1.41 (95% CI: 0.94–2.12, *p* = 0.10) [26]. Stent thrombosis can occur in 2% of patients within 5 days of discontinuing clopidogrel use [27]. The JGES, ASGE, and ESGE guidelines recommend a 5- to 7-day clopidogrel withdrawal period for high-risk endoscopic procedures [14−16]; this case may have been influenced by a longer withdrawal period. In the second case, the patient was on DOAC and underwent CSP after a 2-day withdrawal from DOAC. DOAC administration was resumed the day after treatment; however, the patient developed a transient ischemic attack. The postoperative thromboembolic event rate was low in a meta-analysis with a pooled estimate of 0.41% (95% CI, 0.29–0.54) [28]. The ESGE guidelines suggest a withdrawal period of 2 days for DOACs [16]. In contrast, the JGES and ASGE guidelines recommend that DOACs be withdrawn on the treatment day [14, 15]. In this case, the withdrawal period was shorter than that in the ESGE guidelines and could not prevent disease onset, even if the ESGE guidelines were followed. However, adherence to the JGES or ASGE guidelines might have prevented the onset of the disease. Theoretically, longer withdrawal periods increase the risk of thrombosis; therefore, it is desirable to keep withdrawal periods as short as possible and continue, if possible, if the appropriate withdrawal period is unknown.

This study had several limitations. First, this was a single-center retrospective cohort study, and selection bias was unavoidable. Second, confounding factors were successfully balanced using PSM; however, unmeasured confounding factors could not be balanced between the two groups. Third, the sample size may have been small relative to DPPB as DPPB was rare in this study. Therefore, there may not have been sufficient power. However, as this study was a pilot study, the findings herein can be used in future studies. Finally, immediate bleeding was not assessed. Immediate bleeding has been reported to increase with continuous antithrombotic drug administration [17−19]. Therefore, it was not possible to assess safety regarding immediate bleeding in this study. However, there have been no studies on uncontrolled immediate bleeding with continuous antithrombotic drug use, and no uncontrolled immediate bleeding was observed in the present study. Therefore, it is presumed that immediate bleeding in CSP under continuous antithrombotic drug use may be increased but controllable.

The strength of this study is that it assessed the DPPB rate of CSP under continuous antithrombotic drug administration using RWD. To the best of our knowledge, there have been no such reports till date. This study shows that CSP with continuous antithrombotic drug administration does not increase DPPB rate, and CSP may be recommended in patients on antithrombotic drugs. Thus, in theory, the risk of thromboembolism associated with withdrawal could be avoided, as well as the need for repeated colonoscopy, which increases costs and is burdensome for both patients and doctors.

In conclusion, as per RWD, CSP with continuous antithrombotic drug administration did not significantly increase DPPB rate. Therefore, for patients receiving antithrombotic drugs, CSP with continuous antithrombotic drug administration can be a safe and simple treatment option.

## Statement of Ethics

This study was approved by the Toranomon Hospital Ethics Committee (approval number: 2313) and conducted in accordance with the 1975 Declaration of Helsinki (6th edition, 2008). Informed consent was obtained from the patients through an opt-out method. The need for informed consent was waived by the Toranomon Hospital Ethics Committee.

## Conflict of Interest Statement

The authors have no conflicts of interest to declare.

## Funding Sources

No funding was received for this study.

## Author Contributions

All authors contributed to the study conception and design. Material preparation, data collection, and analysis were performed by Junnosuke Hayasaka, Satoshi Yamashita, Akira Matsui, Kawai Yusuke, Yorinari Ochiai, Takayuki Okamura, Yugo Suzuki, Yutaka Mitsunaga, Kosuke Nomura, Masami Tanaka, Kazuhiro Fuchinoue, Hiroyuki Odagiri, Daisuke Kikuchi, Yutaka Takazawa, and Shu Hoteya. The first draft of the manuscript was written by Junnosuke Hayasaka, and all authors commented on previous versions of the manuscript. All authors read and approved the final manuscript.

## Figures and Tables

**Table 1:** Characteristics of the antithrombotic drug continuation and withdrawal groups

	Continuation group, *N* = 134	Withdrawal group, *N* = 293	*p* value	Continuation group, *N* = 116	Withdrawal group, *N* = 116	*p* value	Absolute standardized difference
Age	72.8±8.8	73.5±7.8	0.40	73.5±7.8	73.6±7.4	0.97	0.006
Male	106 (79.1)	245 (83.6)	0.32	92 (79.3)	92 (79.3)	1.00	<0.001
Charlson comorbidity index	3.00 [1.00, 6.00]	2.00 [1.00, 4.00]	0.094	2.50 [1.00, 6.00]	3.00 [1.00, 5.00]	0.73	0.012
Antiplatelet drug	103 (76.9)	187 (63.8)	0.010	86 (74.1)	83 (71.6)	0.77	0.058
Aspirin	78 (58.2)	118 (40.3)	0.001	61 (52.6)	61 (52.6)	1.00	<0.001
Thienopyridine	21 (15.7)	51 (17.4)	0.76	21 (18.1)	17 (14.7)	0.60	0.093
Cilostazol	7 (5.2)	35 (11.9)	0.047	7 (6.0)	6 (5.2)	1.00	0.037
Anticoagulant	31 (23.1)	122 (41.6)	<0.001	30 (25.9)	33 (28.4)	0.77	0.058
Warfarin	12 (9.0)	14 (4.8)	0.15	11 (9.5)	8 (6.9)	0.63	0.094
DOAC	19 (14.2)	107 (36.5)	<0.001	19 (16.4)	25 (21.6)	0.40	0.13
Fragmin	0 (0.0)	1 (0.3)	1.00	0 (0.0)	0 (0.0)	NA	<0.001
Multiple antithrombotic drug	3 (2.2)	33 (11.3)	0.001	3 (2.6)	1 (0.9)	0.61	0.13
Indication for antithrombotic drug							
Cerebral ischemic disease	28 (20.9)	78 (26.6)	0.25	28 (24.1)	28 (24.1)	1.00	<0.001
Atrial fibrillation	23 (17.2)	103 (35.2)	<0.001	23 (19.8)	25 (21.6)	0.87	0.043
Ischemic heart disease	65 (48.5)	70 (23.9)	<0.001	49 (42.2)	49 (42.2)	1.00	<0.001
Pulmonary embolism or deep vein thrombosis	5 (3.7)	10 (3.4)	1.000	5 (4.3)	6 (5.2)	1.00	0.041
Arteriosclerosis obliterans	3 (2.2)	10 (3.4)	0.76	3 (2.6)	2 (1.7)	1.00	0.059
Valvular disease	5 (3.7)	4 (1.4)	0.15	2 (1.7)	2 (1.7)	1.00	<0.001
Others	6 (4.5)	28 (9.6)	0.11	6 (5.2)	4 (3.4)	0.75	0.085
Treatment at hospitalization	63 (47.0)	109 (37.2)	0.070	49 (42.2)	41 (35.3)	0.35	0.14
Scheduled treatment	49 (36.6)	130 (44.4)	0.16	44 (37.9)	38 (32.8)	0.49	0.11
Expert endoscopist	105 (78.4)	243 (82.9)	0.32	94 (81.0)	91 (78.4)	0.74	0.064
Clipping	88 (65.7)	197 (67.2)	0.84	75 (64.7)	79 (68.1)	0.68	0.073

Values are presented as *n* (%), median [interquartile range], or mean ± standard deviation. Statistical significance was set at *p* < 0.05. Thienopyridine included clopidogrel, prasugrel, and ticlopidine. The DOAC included apixaban, dabigatran, edoxaban, and rivaroxaban. Others included Aberdeen varicose veins, atrioventricular block, eosinophilic granulomatosis with polyangiitis, essential thrombocythemia, hemorrhage in the ocular fundus, polycythemia, prophylactic administration, protein S deficiency, shunt obstruction, and Takayasu arteritis. DOAC, direct oral anticoagulants; NA, not applicable.

**Table 2:** Characteristics of polyps in each group

	Continuation group, *N* = 134	Withdrawal group, *N* = 293	*p* value
Total number of polyps	266	683	
Mean number of polyps per patient	2.0 [1.0, 2.8]	2.0 [1.0, 3.0]	0.21
Preoperative lesion size	5.4±1.7	5.4±1.7	0.91
Specimen size	7.5±3.6	7.1±3.4	0.14
Location			
Right-sided colon (vs. left-sided colon and rectum)	164 (61.7)	439 (64.3)	0.50
Morphology			
Polypoid type (vs. non-polypoid)	212 (79.7)	510 (75.7)	0.22
Pathological diagnosis			
High-grade adenoma or cancer (vs. low-grade adenoma or other benign polyps)	14 (5.3)	28 (4.1)	0.56

Values are *n* (%), median [interquartile range], or mean ± standard deviation. *p* value <0.05 was considered to indicate statistical significance.

**Table 3:** Number of days of antithrombotic therapy and of days between treatment and resumption

	Withdrawal	Resumption
Antiplatelet		
Aspirin	5 [3, 7]	1 [1, 1]
Thienopyridine	7 [7, 10]	1 [1, 1]
Cilostazol	2 [2, 5]	1 [1, 1]
Anticoagulant		
Warfarin	5 [3, 6]	1 [1, 1]
DOAC	1 [1, 2]	1 [1, 1]
Fragmin	1 [1, 1]	1 [1, 1]

Values are median [interquartile range]. DOAC, direct oral anticoagulant.

**Table 4:** Comparison of outcomes before and after PSM between both groups

	Before PSM			After PSM		
	continuation group, *N* = 134	withdrawal group, *N* = 293	*p* value	continuation group, *N* = 116	withdrawal group, *N* = 116	*p* value
Primary outcome						
Delayed bleeding	2 (1.5)	1 (0.3)	0.23	1 (0.9)	0 (0.0)	1.00
Secondary outcomes						
Minor bleeding	0 (0.0)	1 (0.3)	1.00	0 (0.0)	0 (0.0)	NA
Consultation	0 (0.0)	1 (0.3)		0 (0.0)	0 (0.0)	
Hospitalization	0 (0.0)	0 (0.0)		0 (0.0)	0 (0.0)	
Colonoscopy	0 (0.0)	0 (0.0)		0 (0.0)	0 (0.0)	
Thromboembolism	0 (0.0)	2 (0.7)	1.00	0 (0.0)	0 (0.0)	NA
Perforation	0 (0.0)	0 (0.0)	NA	0 (0.0)	0 (0.0)	NA

Values are *n* (%). *p* value <0.05 was considered to indicate statistical significance.

NA, not applicable.
